# Management of mild degenerative cervical myelopathy and asymptomatic spinal cord compression: an international survey

**DOI:** 10.1038/s41393-023-00945-8

**Published:** 2023-12-21

**Authors:** Jamie F. M. Brannigan, Benjamin M. Davies, Oliver D. Mowforth, Ratko Yurac, Vishal Kumar, Joost Dejaegher, Juan J. Zamorano, Rory K. J. Murphy, Manjul Tripathi, David B. Anderson, James Harrop, Granit Molliqaj, Guy Wynne-Jones, Jose Joefrey F. Arbatin, So Kato, Manabu Ito, Jefferson Wilson, Ronie Romelean, Nicolas Dea, Daniel Graves, Enrico Tessitore, Allan R. Martin, Aria Nouri

**Affiliations:** 1 Division of Neurosurgery, Department of Clinical Neurosciences, Addenbrooke’s Hospital, University of Cambridge, Cambridge, UK; 2 School of Clinical Medicine, University of Cambridge, Cambridge, UK; 3 Spine unit, Department of Orthopedic and Traumatology, Clínica Alemana, Santiago, Chile; 4 Department of Orthopedic and Traumatology, School of Medicine, University del Desarrollo, Santiago, Chile; 5 Department of Orthopaedics, PGIMER, Chandigarh, India; 6 Department of Neurosurgery, University Hospitals Leuven, Leuven, KU Leuven Belgium; 7 Department of Neurosurgery, St. Joseph’s Hospital and Medical Center, Barrow Neurological Institute, Phoenix, Arizona USA; 8 Faculty of Medicine and Health, University of Sydney, Sydney, Australia; 9 Department of Neurological Surgery, Thomas Jefferson University, Philadelphia, PA USA; 10 Division of Neurosurgery, Geneva University Hospitals, Geneva, Switzerland; 11 The Newcastle Upon Tyne Hospitals NHS Foundation Trust, Newcastle, UK; 12 Orthopedic surgeon, Spine and Orthopedics, Chong Hua Hospital, Cebu, Philippines; 13 The University of Tokyo Hospital, Tokyo, Japan; 14 Department of Orthopaedic Surgery, National Hospital Organization Hokkaido Medical Center, Sapporo, Japan; 15 Division of Neurosurgery, Department of Surgery, University of Toronto, Toronto, ON Canada; 16 Jayapalan Division of Neurosurgery, Department of Surgery, University Malaya Medical Centre, Petaling Jaya, Kuala Lumpur Malaysia; 17 Combined Neurosurgical and Orthopedic Spine Program. Vancouver General Hospital, University of British Columbia, Vancouver, Canada; 18 University of California, Davis, California, USA

**Keywords:** Spinal cord diseases, Spinal cord diseases

## Abstract

**Study design:**

Cross-sectional survey.

**Objective:**

Currently there is limited evidence and guidance on the management of mild degenerative cervical myelopathy (DCM) and asymptomatic spinal cord compression (ASCC).

Anecdotal evidence suggest variance in clinical practice. The objectives of this study were to assess current practice and to quantify the variability in clinical practice.

**Methods:**

Spinal surgeons and some additional health professionals completed a web-based survey distributed by email to members of AO Spine and the Cervical Spine Research Society (CSRS) North American Society. Questions captured experience with DCM, frequency of DCM patient encounters, and standard of practice in the assessment of DCM. Further questions assessed the definition and management of mild DCM, and the management of ASCC.

**Results:**

A total of 699 respondents, mostly surgeons, completed the survey. Every world region was represented in the responses. Half (50.1%, *n* = 359) had greater than 10 years of professional experience with DCM. For mild DCM, standardised follow-up for non-operative patients was reported by 488 respondents (69.5%). Follow-up included a heterogeneous mix of investigations, most often at 6-month intervals (32.9%, *n* = 158). There was some inconsistency regarding which clinical features would cause a surgeon to counsel a patient towards surgery. Practice for ASCC aligned closely with mild DCM. Finally, there were some contradictory definitions of mild DCM provided in the form of free text.

**Conclusions:**

Professionals typically offer outpatient follow up for patients with mild DCM and/or asymptomatic ASCC. However, what this constitutes varies widely. Further research is needed to define best practice and support patient care.

## Introduction

Degenerative cervical myelopathy (DCM) is an umbrella term for symptomatic spinal cord compression, secondary to degenerative changes of the cervical spine [1–3]. Symptoms are often debilitating and progressive, typically resulting in permanent disability and poor quality of life [4–8].

Disease severity is often quantified using the modified Japanese Orthopaedic Association (mJOA) score, an objective physician evaluation of neurological dysfunction [9]. Moderate and severe DCM are defined as cases where mJOA ≤14 [10] and surgery has been recommended for such patients, as outlined in the most recent clinical practice guidelines [11]. Prior to surgery, many factors contribute to a delay in diagnosis [12, 13] in moderate/severe DCM. Nonetheless, there is a consensus amongst surgeons in support of operative management.

In contrast, there is weak evidence informing the management of mild DCM [11], most commonly defined in the literature as mJOA 15–17 [10]. Amongst mild DCM patients, there is a subpopulation that deteriorates and another which remains stable [14]. An absence of prognostic biomarkers and symptoms [15] means that there are no strong predictions to support decision making.

Guidelines therefore currently suggest surgical intervention or a trial of structured rehabilitation in mild DCM, with the latter escalating to the former in cases of deterioration [11]. This tentative guidance is reflected in variable practice, as has been anecdotally reported by surgeons. There is no specification on what structured rehabilitation or surveillance should entail [16].

Similar heterogeneity is believed to exist in the management of ASCC, a precursor to DCM [14]. In this case, the guidelines cite weak evidence supporting non-operative management, involving counselling and education [11]. Likewise, what this should constitute remains undefined.

Whilst widespread underdiagnosis [17, 18] so far limits the number of patients with mild or asymptomatic disease in spinal clinics, this is estimated to be vast (one meta-analysis estimates 1 in 5 adults could have ASCC) and rising with aging. Initiatives to accelerate diagnosis are on-going [15, 19], and anticipated to increase case ascertainment.

On this background of weak evidence and variable practice, and driven by projected demand, efforts to better inform care for mild DCM and ASCC are a critical priority. The objective of this study was to assess current practices in the assessment and management of mild DCM and ASCC. This work also aligns with two other research priorities of the AO Spine RECODE-DCM initiative [20]: *diagnostic criteria* [19] and *assessment and monitoring* [21]. We hypothesise that there is substantial variability in the definition, assessment, and management of mild DCM and ASCC.

## Methods

The survey is reported following the Checklist for Reporting Results of Internet E-Surveys [22] (CHERRIES).

### Survey design

A cross-sectional observational study was conducted utilising a web-based survey targeted at surgeons who operate on the cervical spine, along with physicians and allied health professionals (AHPs) involved in the DCM patient journey.

The survey questions can be found in Supplementary Material 1. Questions captured experience with DCM, frequency of DCM patient encounters, and standard of practice in the assessment of DCM. Further questions assessed the definition and management of mild DCM, and the management of asymptomatic spinal cord compression.

Question format was a mostly of multiple-choice questions, the only exception being a question requiring a full-text answer.

### Ethical approval and consent

The survey was approved by AO Spine before dissemination amongst the surgeon community.

Participants completed the survey voluntarily and were informed before doing so that anonymised data would be shared with researchers associated with the AO Spine Knowledge Forum Spinal Cord Injury for the purposes of academic research.

This acted as voluntary electronic consent, with completion of the survey questions taken as agreement to participate.

### Development and testing

The usability and technical functionality of the survey was piloted by a team of spinal surgeons from the AO Spine Natural History Incubator [23] before dissemination.

### Data protection

No patient identifiable information was stored. The minimum amount of data was securely stored and accessed by the minimum number of researchers for the minimum amount of time required to complete the research.

### Participants

All participants were practicing surgeons, physicians, AHPs or academics, based in centres around the world.

### Recruitment

An open survey type was utilised. Surgeons were recruited to a web-based questionnaire, administered by SurveyMonkey (Momentive, California, USA). The survey was disseminated via email directly to the members of AO Spine. In addition, a request to submit the survey to the CSRS North American society was undertaken and accepted.

No contact was made with participants outside of the survey.

### Administration

AO Spine is a not-for-profit institution, comprising the world’s largest community of spinal surgeons, researchers and allied spine professions. There was no sample pre-selection. The survey was administered via email. Completion of the survey was voluntary, and no incentives were offered. Respondents were able to review their answers by using a “Back” button. Responses were collected from 12th October 2021 to July 7th 2022.

### Response rates

In 2021, AO Spine consisted of approximately 6000 members. As 688 participants from AO Spine completed the survey, the theoretical minimum response rate was 11%. The completion rate was calculated by SurveyMonkey to be 78%. After the initial email drive via AO Spine, a low response rate from North America was observed, and therefore a formal request to survey the CSRS North America community was undertaken and accepted.

IP addresses were recorded as metadata with each survey response, allowing assessment for duplication and preventing multiple entries from the same individual.

### Data analysis

Survey data were extracted into an Excel spreadsheet (Microsoft, California, USA). Analysis and data visualisation were performed using R (v4.0.5; R Core Team, 2020) and RStudio (v1.4.1106; RStudio Team, 2021). Incomplete responses were excluded from the analysis, except in cases where incomplete questions were independent from those answered.

## Results

**Fig. 1:**
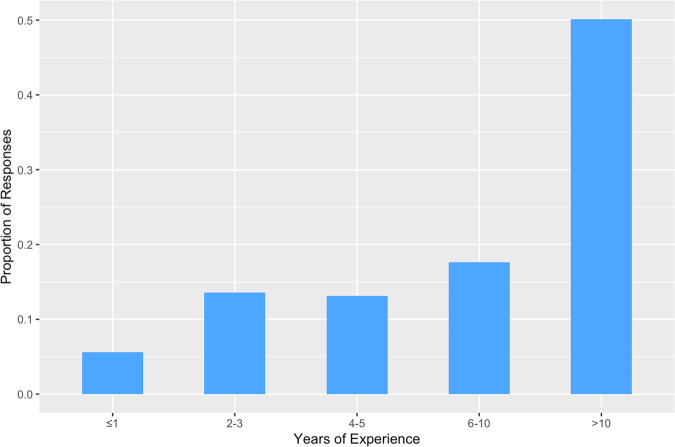
Clinical Experience in Managing Patients with DCM. Bar graph illustrating the proportion of respondents categorised by their years of experience in managing patients with DCM.

A total of 699 responses were received, comprising mostly orthopaedic surgeons (64.0%, *n* = 458) and neurosurgeons (33.2%, *n* = 238), along with a few responses from neurologists/AHPs/academics (2.8%, *n* = 20). The largest proportion of respondents worked in Asia (28.8%, *n* = 206), followed by Latin/South America (24.2%, n = 173), Europe (23.3%, *n* = 167), North America (16.8%, *n* = 120), Africa (2.7%, *n* = 19), the Middle East (2.4%, *n* = 17) and Oceania (2.0%, *n* = 14). A majority of respondents (50.14%, *n* = 359) had greater than 10 years of experience managing patients with DCM (Fig. 1).

**Fig. 2:**
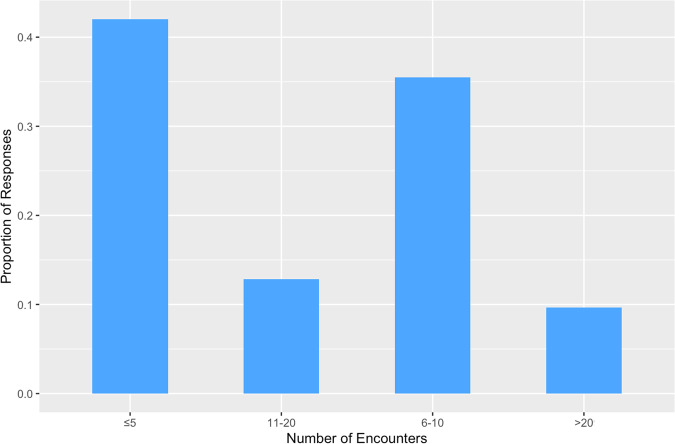
Monthly Clinical Encounters with DCM Patients. Bar chart illustrating the monthly frequency at which respondents encounter patients with DCM.

Most respondents (58.0%, *n* = 415) reported more than 6 encounters with DCM patients each month (Fig. 2).

The full dataset can be found in Supplementary Material 2.

### DCM investigations

In addition to MRI, respondents most often request lateral and AP cervical X-rays (70.3%, 503; Table 1). Only 30 respondents (4.2%) reported using no further investigations when working up a suspected DCM diagnosis.

**Table 1:** Further investigations for suspected DCM.

Investigation	Responses
Lateral and AP cervical X-ray	70.3% (*n* = 503)
Flexion and extension X-ray	64.5% (*n* = 462)
Standing and whole-body X-ray	9.4% (*n* = 67)
Cervical CT scan	48.3% (*n* = 346)
Electrophysiology examination	26.7% (*n* = 191)
Flexion and extension MRI	9.9% (*n* = 71)
Other	7.1% (*n* = 51)
None of the above	4.2% (*n* = 30)

Electrophysiology was mostly reported to be used “rarely/specific cases” (54.5%, *n* = 390) in the diagnosis of DCM (Supplementary Material 3).

### Non-operative patient follow-up

A process of standardised follow-up for non-operative patients was reported by 488 respondents (69.5%).

**Fig. 3:**
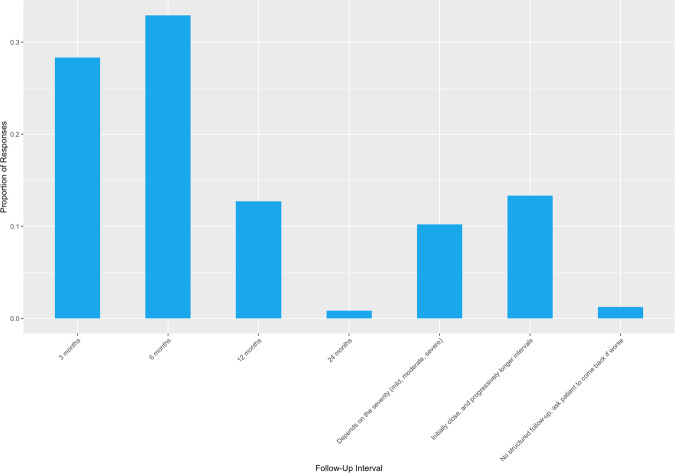
Follow-Up Intervals in Non-Operative DCM Management. Bar chart presenting the proportion of  respondents according to their reported intervals of standardised follow-up in the non-operative management of DCM.

The period of follow-up, where reported, was most often 6 months (32.9%, *n* = 158) or 3 months (28.3%, *n* = 136), as shown in Fig. 3.

**Fig. 4:**
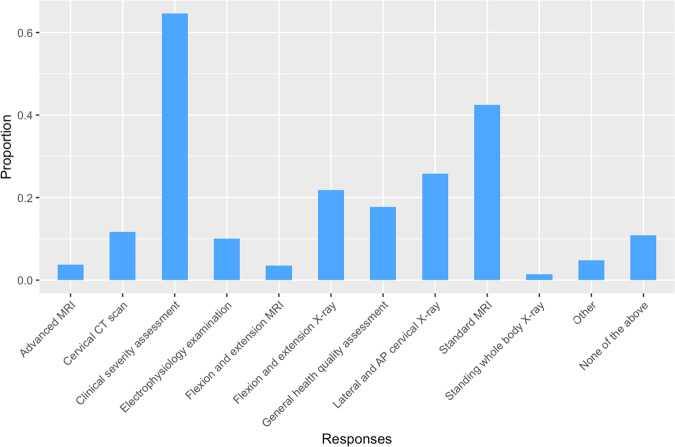
Assessments in Non-Operative DCM Follow-Up. Bar chart illustrating the proportion of respondents employing various assessments during follow-up visits in the non-operative management of DCM.

At follow-up, a clinical severity assessment (e.g., mJOA) was the most frequently reported assessment (64.6%, *n* = 310), in addition to a physical exam (Fig. 4). Approximately half of the respondents reported some form of MRI imaging (49.7%, *n* = 239). Nothing more than a physical exam was reported in 10.6% of cases (*n* = 51).

### Mild DCM definition

When asked to define mild DCM, the free text responses were used to generate a word cloud (Supplementary Material 4).

**Fig. 5:**
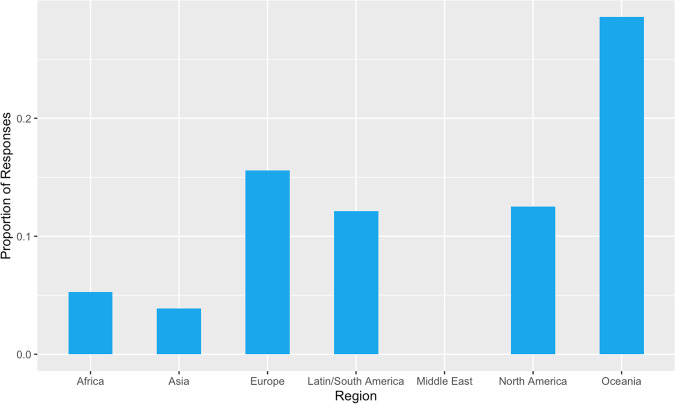
Regional Alignment of mJOA-based mild DCM definitions with recent guidelines (15–17). Bar chart displaying the proportion of mJOA-based mild DCM definitions in alignment with recent guidelines across different world regions.

Many respondents opted for a definition based upon the mJOA score. An mJOA score of 15–17 was most commonly cited as the definition of mild DCM, where a score was specified (46.2%, *n* = 49). A variation of this answer, with no upper bound (mJOA≥15), was provided as a definition by 25 respondents (23.6% of those using an mJOA-based definition). A geographical subgroup analysis of mild DCM definitions was performed (Fig. 5). Each value represents the proportion of mJOA-based score definitions, per region. The greatest proportion of mJOA score definitions aligned with the international guidelines were provided by respondents in Oceania (28.6%, *n* = 4). Of the 17 respondents in the Middle East, no such mJOA definitions were provided.

Score ranges unrelated to the guidelines (e.g., mJOA >12, 14–15) were provided by 32 respondents.

The remaining responses used discrete clinical and imaging findings as a proposed definition.

### Mild DCM management

**Fig. 6:**
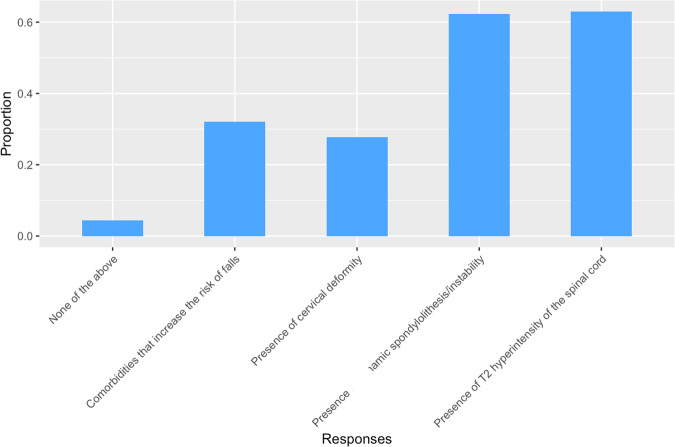
Factors Influencing Surgical Recommendation in Mild DCM. Bar chart illustrating the proportion of respondents' opinions on various factors that would influence their decision to recommend surgery for patients with mild DCM.

When asked what factors would influence a decision to recommend surgery to mild DCM patients, most responses included ‘presence of T2 hyperintensity of the spinal cord’ and/or “presence of dynamic spondylolisthesis/instability” (Fig. 6).

If non-operative management was pursued in a case of mild DCM, there was variation in the changes that would prompt a recommendation of surgery (Table 2).

**Table 2:** Changes that would prompt a recommendation of surgery.

Findings	Responses
Deterioration following recent trauma	75.83% (*n* = 436)
The presence of instability/spondylolisthesis evident on dynamic imaging	64.2% (*n* = 369)
Minor worsening of neurological exam (e.g., loss of 1 point on mJOA/JOA)	60.2% (*n* = 346)
Progression of cervical kyphosis/deformity	57.9% (*n* = 333)
Worsening identified by relatives/carers	53.0% (*n* = 315)
No change in neurological exam but the patient subjectively feels worse	47.3% (*n* = 272)
No change in neurological status but patient indicates accidental falls since the last consultation	39.5% (*n* = 227)
The patient does not wish to have lifestyle restrictions (e.g., participation in contact sports)	29.7% (*n* = 171)
Other comorbidities that increase the risk of falls (e.g., Parkinson’s disease)	26.8% (*n* = 154)
The patient remains impaired but has improved since the last follow-up	4.5% (*n* = 26)
No change in neurological exam and the patient remains subjectively stable	1.9% (*n* = 11)
None of the above	1.0% (*n* = 6)

### ASCC assessment and management

Similar questions were posed in the context of ASCC.

**Fig. 7:**
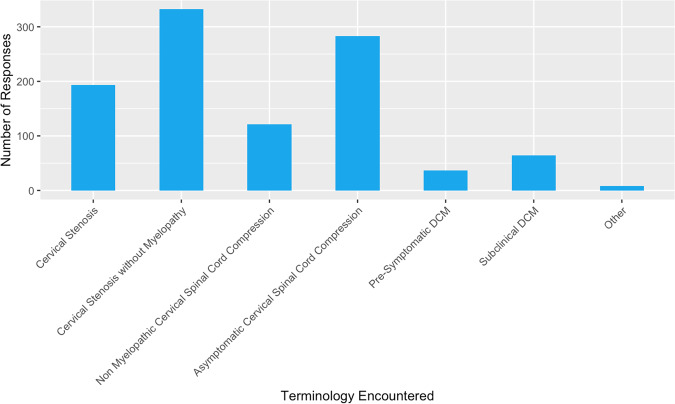
Clinical Terminology for Asymptomatic Spinal Cord Compression. Bar chart showing the frequency of terms encountered in clinical practice that refer to asymptomatic spinal cord compression.

The most common descriptive term used clinically for ASCC was reported as ‘cervical stenosis without myelopathy’ (58.17%, *n* = 331), followed by ‘asymptomatic cervical spinal cord compression’ (49.56%, *n* = 282; Fig. 7).

**Fig. 8:**
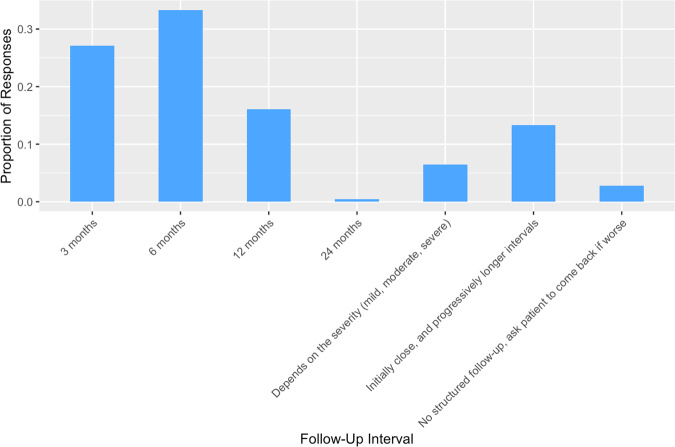
Follow-Up Intervals for Asymptomatic Spinal Cord Compression Management. Bar chart displaying the standard follow-up intervals as reported by respondents for managing asymptomatic spinal cord compression.

A standardised follow-up schedule was employed by 588 respondents (75.17%). The interval of follow-up (Fig. 8) and additional assessments (Supplementary Material 5) mapped closely to responses in the context of mild DCM.

## Discussion

Our results suggest that there is no consensus on the management of either mild DCM or ASCC. Moreover, responses indicate that the definition of mild DCM is uncertain and that follow-up assessment practices may be deficient. Moreover, the terminology used in the context of ASCC is mixed. Regarding international comparisons, heterogeneity was identified within each region surveyed, suggesting that the variation observed exists at a local level.

### Variability in practice reflects weak evidence informing guidelines

Current international guidelines [11] are tentative and derived from a sparse evidence base. In mild DCM, the guidelines advise surgical intervention or a trial of structured rehabilitation. In ASCC, the guidelines recommend non-operative management, involving counselling and education.

The existence of tentative guidelines was reflected in decisions at several points along the management pathway. At follow-up, no single assessment was performed by more than 67% clinicians, yet 7 different assessments were reported by more than 10% (Fig. 4; Supplementary Material 5). When considering surgery in mild DCM, no decisive clinical feature was reported by more than 75%, however all possible features were reported by more than 30% (Fig. 6).

A more fundamental finding was the varied understanding of the terminology used in the questionnaire. Surprisingly, there was substantial variation in the definition of mild DCM (Supplementary Material 4). It is noteworthy that many reported definitions did not overlap with the objective score-based definition proposed by Tetreault and colleagues [10], and adopted in recent guidelines.

These variations were identified in every region surveyed. For example, standardised follow-up was not provided by between 20%-40% of respondents when divided by region. This suggests that response variation was not due to international cultural factors, and instead more likely due to local factors, such as trust policy and individual decision making.

Together, these findings demonstrate uncertainty amongst clinicians in both diagnosis, definition, and management.

### Inconsistent terminology and practice can delay progress

An inconsistent use of diagnostic terms can give rise to ambiguity when exploring diagnosis, interventions, and outcomes. Further consequences of inconsistency can arise when clinicians are performing literature searches on research databases, or when patients are seeking information related to their condition.

We identified considerable inconsistency in terminology referring to asymptomatic compression of the cervical cord. Every term suggested in our survey had been encountered by respondents (Fig. 7), the most frequent of which were ‘cervical stenosis without myelopathy’ and ‘asymptomatic cervical spinal cord compression’. Whilst not sufficient, consistent disease terminology is a necessary to increase awareness and direct research efforts to better understand asymptomatic compression.

Efforts to establish consensus terminology in DCM provide a template to follow [2, 3] This preceded both an increase in research activity [24] and the publication of international evidence-based clinical practice guidelines [11].

Similarly, over 30% of respondents reported no process for standardised follow-up of non-operative DCM patients, despite best practice recommendations. [11] Equally, at standardised follow-up, over 30% of clinicians reported no use of clinical severity assessments, such as the mJOA score [9]. This implies that non-standardised and/or subjective measures were used by clinicians at follow-up.

Failure by clinicians to use valid, reliable, and responsive outcome measures for DCM limits their capacity to capture the severity of a patient’s condition now, but also over time [25]. Not using valid outcome measures such as the mJOA also provides no interpretable measure of severity for other clinicians. The importance of valid outcome measures is becoming increasingly known, with a series of initiatives such as the Core Outcome Measures in Effectiveness Trials (COMET) increasing in use. The existing lack of priority of outcome measures amongst clinicians may reflect current medical education [26, 27], which is an important component of the number one research priority of the AO Spine RECODE-DCM initiative: *improving awareness* [28].

### Limitations and future work

The survey was principally disseminated by two global spinal organisations; AO Spine and CSRS and it is noted that some groups may have been underrepresented. For example, Asia contributed 29% of responses, and Africa only 3%. Further the survey used a clinician’s recollection of practice, making it vulnerable to recall bias. The overall large sample size, and the broad heterogeneity, with absence of trends event amongst subgroups, suggest this has not limited the findings.

The most pressing future work is to generate robust guidelines for non-operative patients.

This would benefit from a more detailed characterisation of DCM natural history, including the identification of any predictors of deterioration. Standardised patient follow-up is necessary to perform this analysis. A combination of clinician education and practical decision support tools may form first steps to ensure that this is conducted, using expect opinion to bridge known evidence gaps.

## Conclusions

There is a lack of consensus internationally on the management of mild DCM and ASCC. Weak guidelines are informed by a limited understanding of disease natural history. Developing a framework for this is needed both to support patient care, but also enable the evidence to be generated to advance our understanding for the future.

### Supplementary information

Supplementary Materials Summary

Supplementary Material 1

Supplementary Material 2

## Data Availability

Anonymised survey responses can be found in Supplementary Material 2.
