# Supplementary material for: Management of mild degenerative cervical myelopathy and asymptomatic spinal cord compression: an international survey
Source: Spinal Cord. 2023 Dec 21;62(2):51–8. doi: 10.1038/s41393-023-00945-8 (PMC10853067; doi:10.1038/s41393-023-00945-8)

Supplementary Material 1. Survey questions

Data can be found in PDF entitled ‘Supplementary Material 1’.

Supplementary Material 2. Raw anonymised dataset

Data can be found in Excel spreadsheet entitled ‘Supplementary Material 2’.

Supplementary Material 3. Reported frequency of use of electrophysiology investigations in the diagnosis of DCM


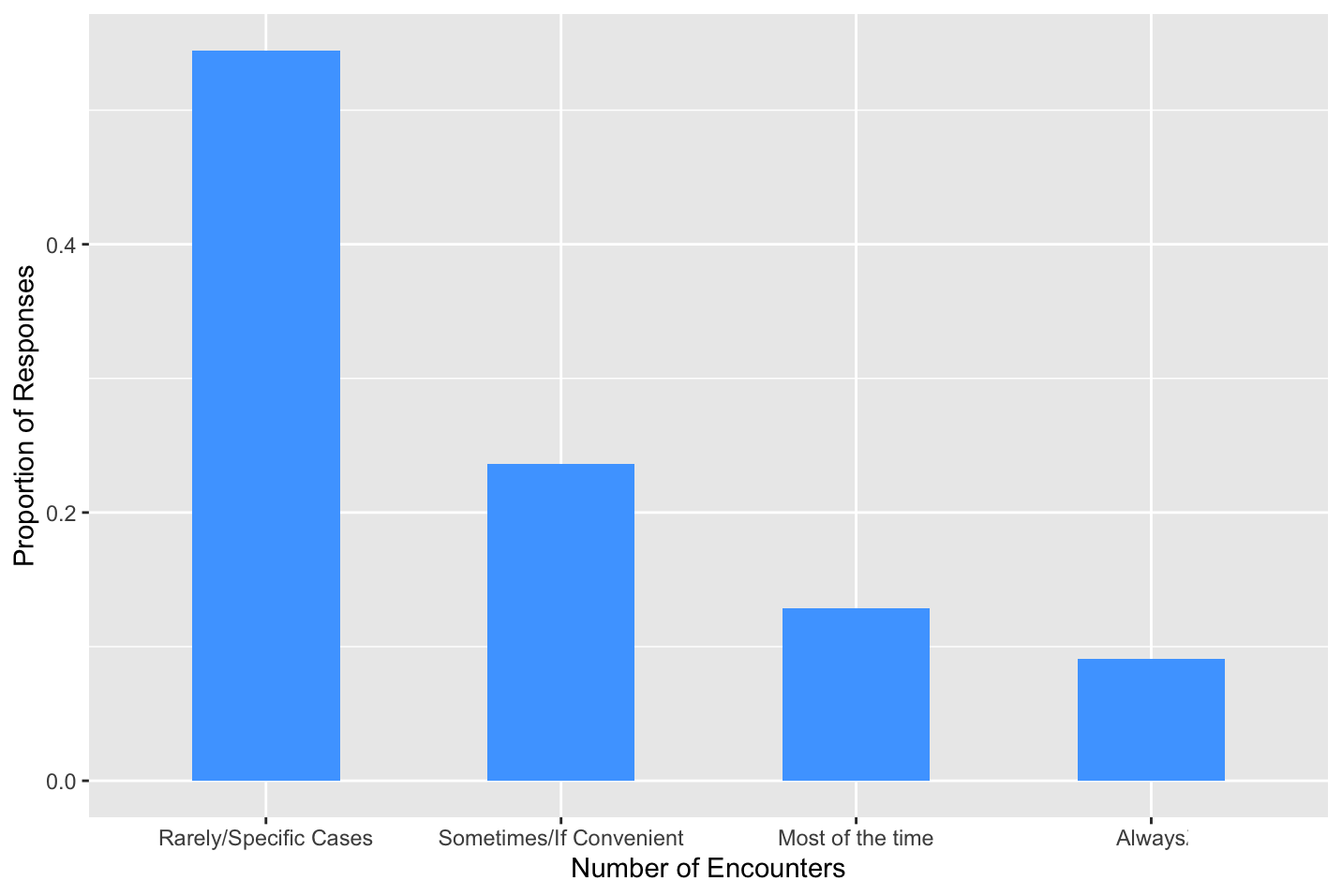


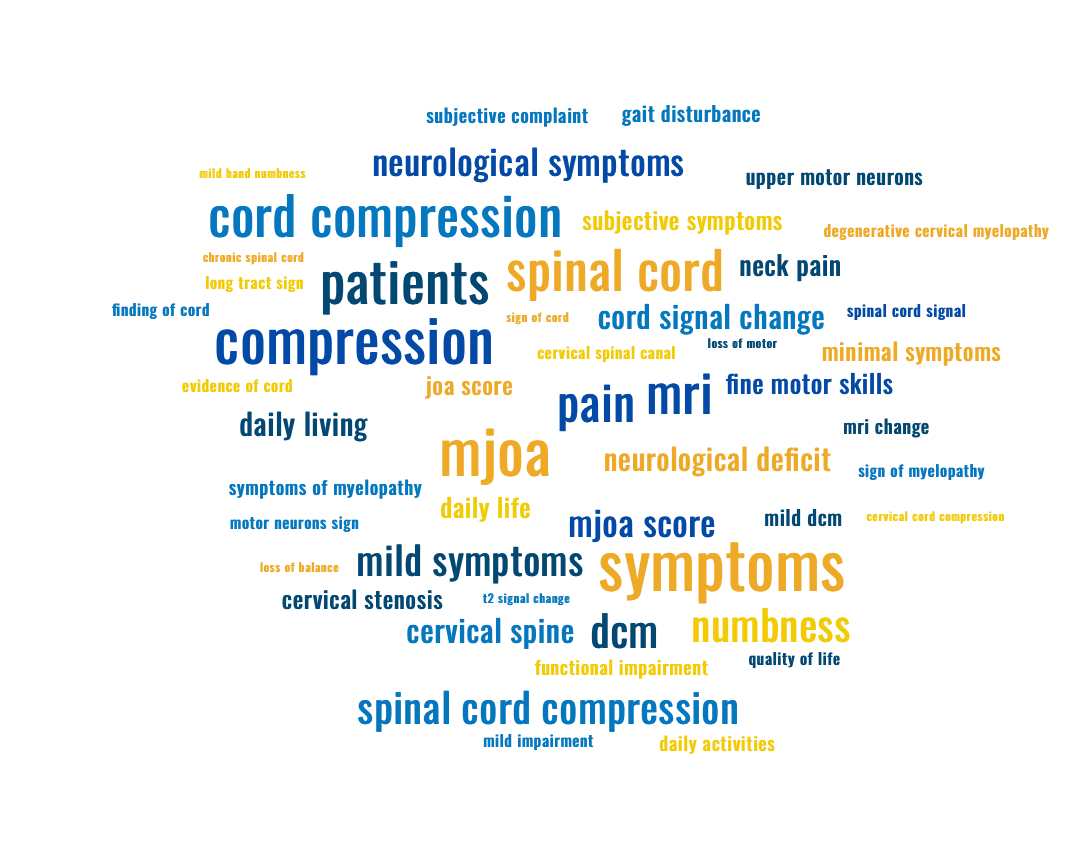
Supplementary Material 4. Word cloud of respondent definition of *mild DCM*

Supplementary Material 5. Additional assessments reportedly performed by respondents in cases asymptomatic spinal cord compression


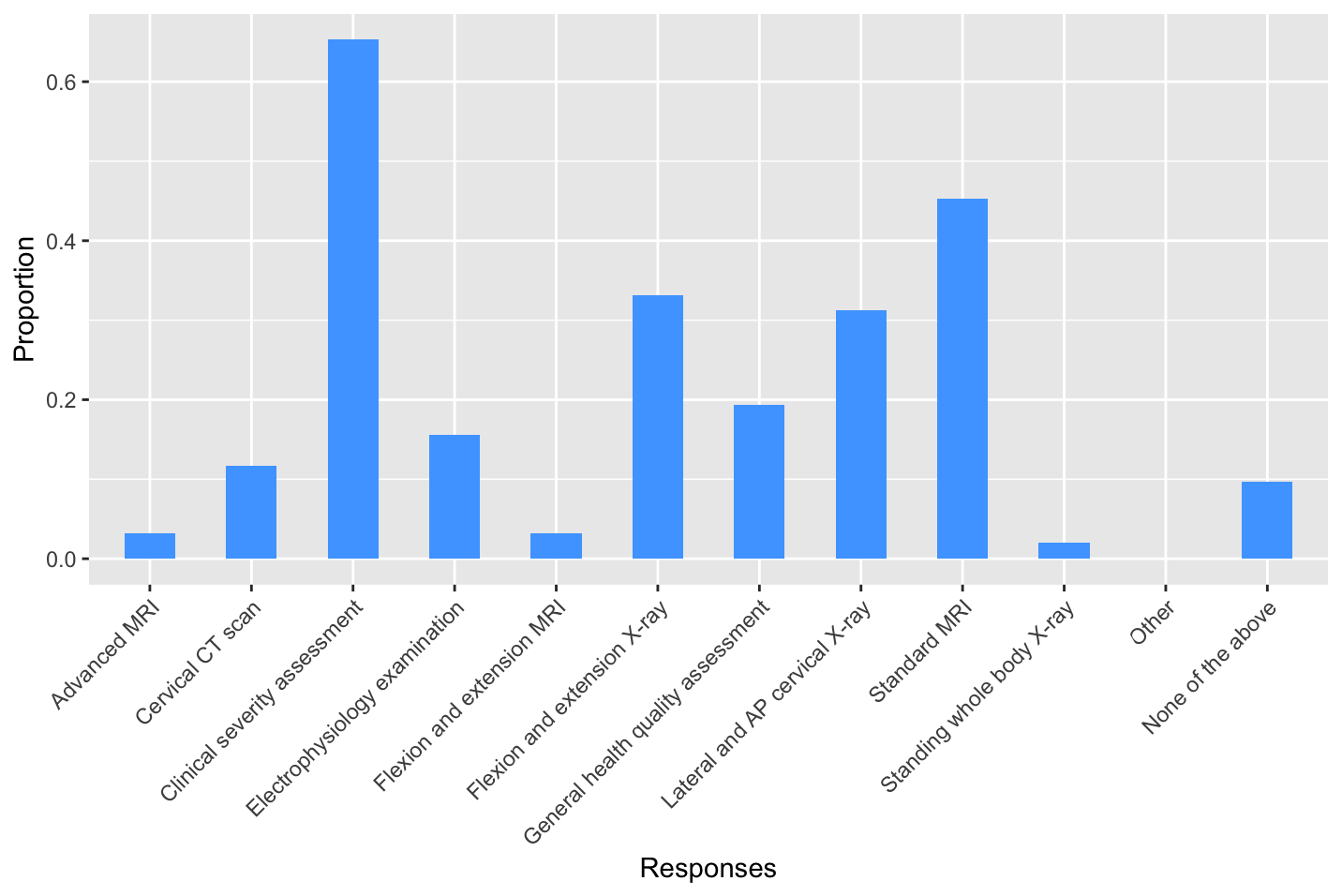

Supplement: Supplementary file 1 — Supplementary Materials Summary [file 41393_2023_945_MOESM1_ESM.docx]
