# Supplementary material for: Management of mild degenerative cervical myelopathy and asymptomatic spinal cord compression: an international survey
Source: Spinal Cord. 2023 Dec 21;62(2):51–8. doi: 10.1038/s41393-023-00945-8 (PMC10853067; doi:10.1038/s41393-023-00945-8)
Supplement: Supplementary file 2 — Supplementary Material 1 [file 41393_2023_945_MOESM2_ESM.pdf]

RECODE DCM

Management of Asymptomatic Spinal Cord Compression: an International Survey

AO Spine RECODE DCM Natural History Incubator

This survey takes approximately 5 minutes, and aims to understand current management of patients with mild DCM who are not initially scheduled for surgery, or patients with asymptomatic spinal cord compression. This was recognised as an important knowledge gap for care by AO Spine RECODE DCM, and now a focus of investigation by its Natural History Incubator.

If you have any questions, or feedback, please contact olesja.hazenbiller@aofoundation.org. For further information about AO Spine RECODE DCM please visit aospine.org/recode

* 1. What best identifies your current position?

- Neurosurgeon
- Orthopaedic Surgeon
- Neurologist
- Neuro/Electrophysiologist
- Physiatrist/Physiotherapist
- Academic/Researcher

* 2. In which region of the world do you work?

- Africa
- Asia
- Europe
- Latin/South America
- Middle East
- North America
- Oceania

* 3. How many years of experience do you have caring for patients with Degenerative Cervical Myelopathy (DCM)?

- ≤1 year
- 2-3 years
- 4-5 years
- 6-10 years
- >10 years

* 4. On average, how many cases of DCM do you see in your practice per month?

- ≤5
- 6-10
- 11-20
- >20

* 5. In a patient that you assess for DCM, which of the following, in addition to MRI do you systematically acquire in the diagnostic process of DCM? Please check all that apply.

- ☐ Lateral and AP cervical X-ray
- ☐ Flexion and extension X-ray
- ☐ Standing whole body X-ray
- ☐ Cervical CT scan
- ☐ Other (please specify: e.g. advanced MRI, gait analysis)
- ☐ None of the above
- ☐ Electrophysiology examination
- ☐ Flexion and Extension MRI

* 6. How often do you use electrophysiology when diagnosing DCM?

Rarely/Specific Cases	Sometimes/If Convenient	Most of the time	Always
○	○	○	○

Management of Asymptomatic Spinal Cord Compression: an International Survey

Non-Operative Management

7. In patients with DCM, who are managed non-operatively (regardless of reason), do you have standardised follow up?

- ○ Yes
- ○ No

Management of Asymptomatic Spinal Cord Compression: an International Survey

Non Operative Management

* 8. At what interval do you follow-up DCM patients in your standardised pathway?

* 9. Besides a physical exam, do you conduct any of the following systematically with each follow up? Please check all that apply.

- [ ] Clinical severity assessment using mJOA, Nurick or other DCM specific measures
- [ ] General health quality assessment (e.g., EQ5D, PROMIS, SF-36)
- [ ] Lateral and AP cervical X-ray
- [ ] Flexion and extension X-ray
- [ ] Standing whole body X-ray
- [ ] Cervical CT scan
- [ ] Other (please specify)
- [ ] None of the above
- [ ] Electrophysiology examination
- [ ] Standard MRI
- [ ] Advanced MRI (e.g., DTI)
- [ ] Flexion and extension MRI

Management of Asymptomatic Spinal Cord Compression: an International Survey

Mild DCM

* 10. Considering the AOSpine guidelines recommendation on patients with mild DCM “offering surgical intervention or a supervised trial of structured rehabilitation for patients with mild DCM”, would any of the following affect your decision more to counsel towards surgery? Please check all that apply.

- [ ] Presence of T2 hyperintensity of the spinal cord
- [ ] Presence of cervical deformity
- [ ] Presence of dynamic spondylolithesis/instability
- [ ] Comorbidities that increase the risk of falls
- [ ] None of the above

* 11. If you follow a patient with mild DCM treated non-operatively, at what point would you counsel the patient towards surgery? Please check all that apply.

- [ ] Minor worsening of neurological exam (e.g., loss of 1 point on mJOA/JOA)
- [ ] No change in neurological exam but the patient subjectively feels worse
- [ ] No change in neurological exam and the patient remains subjectively stable
- [ ] The patient remains impaired but has improved since the last follow-up
- [ ] No change in neurological status but patient indicates accidental falls since the last consultation
- [ ] The patient does not wish to have lifestyle restrictions (e.g., participation in contact sports)
- [ ] Other comorbidities that increase the risk of falls (e.g., Parkinson’s disease)
- [ ] Worsening identified by relatives/carers
- [ ] The presence of instability/spondylolisthesis evident on dynamic imaging
- [ ] Progression of cervical kyphosis/deformity
- [ ] Deterioration following recent trauma
- [ ] None of the above

* 12. What is your definition of mild DCM?

* 13. Do you counsel patients about the risk of traumatic spinal cord injury?

- ○ No
- ○ Yes

RECODE DCM

Management of Asymptomatic Spinal Cord Compression: an International Survey

Regarding Patients with MRI evidence of Asymptomatic Spinal Cord Compression

* 14. If you assess an individual with MRI evidence of Spinal Cord Compression without neurological symptoms, do you employ a standardised follow-up schedule?

- ◯ Yes
- ◯ No

Management of Asymptomatic Spinal Cord Compression: an International Survey

Regarding Patients with MRI evidence of Asymptomatic Spinal Cord Compression

* 15. At what interval do you follow-up these patients in your standardised pathway?

* 16. Besides a physical exam, do you conduct any of the following systematically with each follow up? Please check all that apply.

- [ ] Clinical severity assessment using mJOA, Nurick or other DCM specific measures
- [ ] General health quality assessment (e.g., EQ5D, PROMIS, SF-36)
- [ ] Lateral and AP cervical X-ray
- [ ] Flexion and extension X-ray
- [ ] Standing whole body X-ray
- [ ] Cervical CT scan
- [ ] Electrophysiology examination
- [ ] Standard MRI
- [ ] Advanced MRI (e.g., DTI)
- [ ] Flexion and extension MRI
- [ ] None of the above

RECODE DCM

Management of Asymptomatic Spinal Cord Compression: an International Survey

Terminology

17. Regarding patients with MRI evidence of spinal cord compression without diagnosed DCM, which of the following descriptive terms do you see used

- [ ] Non Myelopathic Cervical Spinal Cord Compression
- [ ] Asymptomatic Cervical Spinal Cord Compression
- [ ] Cervical Stenosis without Myelopathy
- [ ] Other (please specify)
- [ ] Cervical Stenosis
- [ ] Pre-Symptomatic DCM
- [ ] Subclinical DCM

18. Do these terms all mean the same thing and/or confer the same relationship to DCM?

Survey Results (Optional)

19. Please enter your email address if you would like to be contacted with the survey results.

Email Address
